# Progression of pulmonary cysts in Birt-Hogg-Dubé syndrome: longitudinal thoracic computed tomography study with quantitative assessment

**DOI:** 10.1186/s12890-023-02483-8

**Published:** 2023-05-23

**Authors:** Su Min Cho, Eun Jin Chae, Jooae Choe, Sang Min Lee, Jin Woo Song, Kyung-Hyun Do

**Affiliations:** 1 Department of Radiology and Research Institute of Radiology, University of Ulsan College of Medicine, Asan Medical Center, 88 Olympic-ro 43 Gil, Songpa-Gu, Seoul, 05505 Republic of Korea; 2 Department of Pulmonary and Critical Care Medicine, University of Ulsan College of Medicine, Asan Medical Center, 88 Olympic-ro 43 Gil, Songpa-Gu, Seoul, 05505 Republic of Korea

**Keywords:** Birt-Hogg-Dubé (BHD) syndrome, Pulmonary cyst, Computed tomography, Pulmonary function test, Pneumothorax, Folliculin gene mutation

## Abstract

**Background:**

Birt-Hogg-Dubé (BHD) syndrome is a rare autosomal dominant disorder characterized by fibrofolliculomas, renal tumors, pulmonary cysts, and recurrent pneumothorax. Pulmonary cysts are the cause of recurrent pneumothorax, which is one of the most important factors influencing patient quality of life. It is unknown whether pulmonary cysts progress with time or influence pulmonary function in patients with BHD syndrome. This study investigated whether pulmonary cysts progress during long-term follow-up (FU) by using thoracic computed tomography (CT) and whether pulmonary function declines during FU. We also evaluated risk factors for pneumothorax in patients with BHD during FU.

**Methods:**

Our retrospective cohort included 43 patients with BHD (25 women; mean age, 54.2 ± 11.7 years). We evaluated whether cysts progress by visual assessment and quantitative volume analysis using initial and serial thoracic CT. The visual assessment included the size, location, number, shape, distribution, presence of a visible wall, fissural or subpleural cysts, and air-cuff signs. In CT data obtained from a 1-mm section from 17 patients, the quantitative assessment was performed by measuring the volume of the low attenuation area using in-house software. We evaluated whether the pulmonary function declined with time on serial pulmonary function tests (PFT). Risk factors for pneumothorax were analyzed using multiple regression analysis.

**Results:**

On visual assessment, the largest cyst in the right lung showed a significant interval increase in size (1.0 mm/year, *p* = 0.0015; 95% confidence interval [CI], 0.42–1.64) between the initial and final CT, and the largest cyst in the left lung also showed significant interval increase in size (0.8 mm/year, *p* < 0.001, 95% CI; -0.49–1.09). On quantitative assessment, cysts had a tendency to gradually increase in size. In 33 patients with available PFT data, FEV1pred%, FEV1/FVC, and VCpred% showed a statistically significant decrease with time (*p* < 0.0001 for each). A family history of pneumothorax was a risk factor for the development of pneumothorax.

**Conclusions:**

The size of pulmonary cysts progressed over time in longitudinal follow-up thoracic CT in patients with BHD, and pulmonary function had slightly deteriorated by longitudinal follow-up PFT.

## Background

Birt-Hogg-Dubé (BHD) syndrome is a rare hereditary disease caused by mutations in the tumor suppressor gene *FLCN* coding for folliculin. The folliculin gene is known to contribute to BHD syndrome by being involved in the mammalian target of rapamycin complex 1 (mTORC1) signaling pathway, but the exact mechanism is still unknown [1, 2]. BHD syndrome is characterized by the development of cutaneous fibrofolliculoma, renal tumors of various histological types, multiple pulmonary cysts, and recurrent pneumothorax [3–5]. Renal cell carcinoma is the most important factor in determining the prognosis of patients with BHD syndrome; however, recurrent pneumothorax in these patients is also related to their quality of life, so it is an important factor when determining their prognosis [6].

Multiple pulmonary cysts are the most common manifestation seen in most patients with BHD syndrome [3]. The morphological characteristics and differential diagnosis of other cystic diseases have been well described [7–9]. Pulmonary cysts are characterized by lower lobe and peripheral lung predominance and air-cuff signs surrounding the adjacent bronchovascular bundle [10, 11]. Until recently, data on the longitudinal change in pulmonary cysts in BHD syndrome were limited to a small series [12], but whether pulmonary cysts progress in size with time is unknown [13–15].

The development of pneumothorax is associated with underlying lung diseases such as chronic obstructive pulmonary disease, previous history of pneumothorax, male sex, degree of emphysema, and smaller total lung capacity (TLC) [16]. Spontaneous pneumothorax is an important and recurrent manifestation of pulmonary involvement in patients with BHD syndrome [6]. A study of 89 families with BHD syndrome reported that the presence or absence of lung cysts, the total lung cyst volume, and the diameter and volume of the largest cyst were associated with pneumothorax in patients with BHD syndrome [17]. Another recent study showed that the diameters and volumes of the largest cysts were associated with the occurrence of pneumothorax in patients with BHD syndrome [18].

It is not known whether pulmonary cysts in patients with BHD syndrome progress over time and whether pulmonary function declines during follow-up. A recent study showed slightly increased residual volume (RV) and reduced diffusing capacity of the lung for carbon monoxide (DLco) over a follow-up period of 6 years [19]. However, the progression of pulmonary cysts has not been studied yet.

This study investigated whether pulmonary cyst progression occurs during the long-term follow-up of patients with BHD syndrome using thoracic CT and whether changes in pulmonary function occur during the follow-up using the pulmonary function test (PFT). We also evaluated risk factors for pneumothorax in patients with BHD during follow-up.

## Methods

### Patients

We conducted a single-center retrospective study of patients diagnosed with BHD syndrome. This retrospective study received approval from the institutional review board of the Asan Medical Center and a waiver for the requirement for informed consent (S2021-0287–0001). The inclusion criteria were a diagnosis of BHD syndrome and at least one follow-up thoracic CT over 6 months. For the diagnosis, we used the diagnostic criteria of BHD syndrome suggested by the European BHD consortium [3]. We found 66 patients presumed to have BHD syndrome by searching the electronic medical record (EMR) system using diagnostic data of BHD and *FLCN* gene test results. We also included 20 patients who were diagnosed with BHD syndrome in the Pulmonology Department and referred by a pulmonologist (J.W.S.). Twenty patients overlapped between the EMR and pulmonology clinic. Among a total of 66 patients from the EMR search and pulmonology cohort, 23 patients were excluded—17 because of diagnostic uncertainty and 6 because of the unavailability of data on follow-up thoracic CT for more than 6 months. Finally, a total of 43 patients were enrolled, and demographic, clinical, genetic, and lung function data were collected from the EMR. Among 43 patients, five patients had alleged family history of BHD syndrome and those five patients were all index patients of their families.

### Visual assessment of pulmonary cysts on thoracic CT

Thoracic CT scans obtained in patients during outpatient clinics and/or admission if patients showed pneumothorax. The mean follow-up interval of thoracic CT in this study was 6.1 years (minimum of 0.6 years. maximum of 14.8 years). A thoracic CT of all patients was obtained in the supine position with full inspiratory status. In cases of work up for pulmonology outpatients, thoracic CT was obtained with full inspiration and expiration in the supine and prone positions. Patients were scanned using a 64- or 128-channel multi-detector computed tomography scanner (Somatom Definition, Definition AS + , and Somatom Definition Flash, Siemens Healthcare), and the scan parameters were as follows: 120–140 kVp, 100 mAs effective with dose modulation, pitch of 1.0, and 16 × 0.75 mm collimation with 0.75 mm thickness. The scan range was from above the clavicle to the level of both adrenal glands. The images were reconstructed at 3-mm intervals without a gap (B50f kernel) and 1-mm intervals with a gap (5-mm gap, B60f kernel) with the axial and coronal planes. All images were viewed at a PACS workstation. The window settings were center = -750 and width = 1500, respectively.

Thoracic CT was evaluated by a chest radiologist with 20 years of experience (E.J.C.) and a radiology resident (S.M.C.). In cases of discrepancies between the two readers, they resolved differences through discussions, arriving at a consensus. On the initial thoracic CT and last thoracic CT, the largest cyst diameter, smallest cyst diameter, their location, number, and shape of the largest cyst, visibility of the cyst walls, distribution, presence of fissural cysts or subpleural cysts, whether the cysts encircled the bronchovascular bundle (air-cuff sign) [10], presence of interstitial abnormality (ground-glass opacity (GGO), reticulation), and presence of emphysema were evaluated. In the study by Park et al., the air-cuff sign was described for the appearance of pulmonary cyst nearly completely encircling bronchovascular bundle [10]. For the measurement of cyst size and review of other findings, the axial and coronal reformatted CT images were examined.

In addition, the initial and final thoracic CT were reviewed to visually assess the progression of cysts. After comparing the two CTs side-by-side, the extent of the disease was classified as stable or progressive. The evaluation was performed for the parameters including size, number, and shape of cysts. Progression in shape was considered positive if the shape of the cyst changed or became irregular.

The serial follow-up CT was used to evaluate whether pneumothorax occurred, and if it did, the occurrence time and frequency.

### Quantitative assessment of pulmonary cysts on thoracic CT

Quantitative evaluation of pulmonary cysts was performed on 17 patients with the data available from 1-mm thick samples during follow-up thoracic CT for more than 6 months. The change in cyst volume at intervals was evaluated quantitatively using the follow-up CT data. A total of 64 CT exams were performed on 17 patients. The average number of CT exams per patient was 3.7, the average follow-up period was 3.8 years, and the median follow-up period was 3.9 years.

The volume of pulmonary cysts was measured as the low attenuation area (LAA) using in-house software developed for emphysema analysis (AView2010; Coreline Inc, Korea) [20]. In the software, cysts are divided by size and the size of the cluster is measured at 2-mm intervals. Cysts < 2 mm could not be differentiated from noise and were excluded from being measured. Therefore, the volume of cysts was the sum of cysts > 2 mm. The volume of the entire lung was obtained and then the percentage of LAA volume was calculated as the percentage of cyst volume.

### Clinical data collection

Demographic, genetic, clinical, and pulmonary function data were collected from medical records. Age, sex, smoking history, history of pneumothorax, family history of spontaneous pneumothorax within second-degree relatives, skin manifestation, presence of renal tumor, folliculin gene mutation, and family history of BHD syndrome within second-degree relatives were identified.

The PFT was performed according to the guidelines of the American Thoracic Society and European Respiratory Society [21], and the tests performed during the follow-up period were investigated every year. The forced expiratory volume in 1 s (FEV1), ratio of FEV1 to forced vital capacity (FEV1/FVC), vital capacity (VC), ratio of residual volume to total lung capacity (RV/TLC), diffusing capacity of the lung for carbon monoxide (DLco), and ratio of DLco to alveolar volume (DLco/VA) were obtained retrospectively. Follow-up PFT data from February 2010 to March 2021were available for 33 patients.

### Data analysis

For statistical analysis, the SAS version 9.4 program (Cary, NC, USA) was used. To compare the differences between the initial CT and final CT, and between the initial PFT and final PFT, the rate of change per year was analyzed using a paired t-test. To compare the differences between the categorical variables of the initial CT and last CT, exact McNemar’s test was performed by converting each frequency into a percentage. The PFT values according to the follow-up period were investigated for a total of six variables: FEV1pred%, FEV1/FVC, VCpred%, DL_CO_pred%, RV/TLC, and DL_CO_/VApred%. The raw data according to the follow-up period of each variable were plotted, and the average value was plotted through spline by applying this to a mixed linear model. For the quantification results of thoracic CT, dot-plotting was performed to identify the overall trend of change. The risk factors for the occurrence of pneumothorax were analyzed using a binary logistic regression model. A *p*-value < 0.05 was considered statistically significant.

## Results

### Patient characteristics

Demographic and clinical characteristics including the PFT of 43 patients with BHD syndrome are presented in Table 1. The mean age of the patients was 54.2 ± 11.7 years, and 41.9% were male. It was observed that 53.4% of patients had a history of pneumothorax, and the frequency varied from 1 to 5 times. Ten patients (23.3%) suffered pneumothorax once, 5 patients (11.6%) twice, and 7 patients (16.3%) more than three times. A family history of pneumothorax was confirmed in 27.9% of cases.

**Table 1:** Baseline characteristics of the study population

Characteristics		Value
Age (years)		54.2 ± 11.7
Sex	Male	41.9 (18)
	Female	59.1 (25)
Smoking history (pack years)		6.6 ± 10.2
Pneumothorax history		51.2(22)
Pneumothorax number	0	48.8 (21)
	1	23.3 (10)
	2	11.6 (5)
	3	7.0 (3)
	4	7.0 (3)
	5	2.3 (1)
Family history of pneumothorax		27.9 (12)
Initial PFT	FEV1%pred	84.0 ± 13.3
	FEV1/FVC (%)	78.6 ± 7.5
	VC %pred	95.2 ± 14.9
	RV/TLC (%)	30.1 ± 6.4
	DLCO %pred	80.7 ± 11.6
	DLCO/VA (%)	93.2 ± 14.2

The mean ± standard deviation was used for continuous data, and the percentage and frequency were indicated for categorical data

*PFT* pulmonary function test, *FEV1* forced expiratory volume in one second, *FVC* forced vital capacity, *VC* vital capacity, *RV* residual volume, *TLC* total lung capacity, *DLco* carbon monoxide transfer factor, *DLco/VA* carbon monoxide transfer coefficient, *%pred* percentage of predicted value capacity

### Visual assessment of pulmonary cysts on thoracic CT

The mean follow-up interval of thoracic CT in this study was 6.1 years (minimum of 0.7 years. maximum of 14.8 years).

The morphological characteristics of pulmonary cysts in the initial thoracic CT of all 43 patients are presented in Table 2. After measuring the size of the largest cyst in the initial CT and final CT, the rate of change was calculated considering the time interval between the two CTs, which is presented in Table 2. The largest cyst in the right lung showed a significant interval increase in size (1.03 mm/year, p = 0.0015; 95% confidence interval [CI], 0.42–1.64) between the initial and final CT, and the largest cyst in the left lung also showed a significant interval increase in size (0.82 mm/year, *p* < 0.001, 95% CI; -0.49–1.09). In addition, the number of patients with air-cuff signs significantly increased on the last CT compared to the initial CT (62.8% to 79.1%, *p* = 0.02).

**Table 2:** Morphologic characteristics of pulmonary cysts on initial CT and temporal changes in cyst characteristics between initial CT and last CT

	**Initial CT**	**Last CT**	**Difference**	***p*-value**
Largest cyst (right lung)	25.1 ± 15.3 mm	26.1 ± 15.5 mm	1.0 (0.42,1.64)	*0.002*
Largest cyst (left lung)	25.0 ± 15.3 mm	25.8 ± 15.6 mm	0.8 (-0.49,1.09)	< *0.001*
Cyst number				0.28
≤ 50	67.4% (29)	60.5% (26)		
50–100	25.6% (11)	27.9% (12)		
≥ 100	7.0% (3)	11.6% (5)		
Cyst shape				0.50
Round/ovoid	14.0% (6)	9.3% (4)		
Lentiform	0.0 (0)	0.0 (0)		
Irregular	86.0% (37)	90.73% (39)		
Septated	0.0% (0)	0.0% (0)		
Location of largest cyst				1.00
Subpleural	83.7% (35)	79.1% (33)		
within lung parenchyma	16.3% (7)	20.9% (9)		
Cyst wall: identifiable	100.0% (43)	100.0% (43)		1.00
Distribution				1.00
Upper	0.0% (0)	0.0% (0)		1.00
Lower	20.9% (9)	20.9% (9)		1.00
Diffuse	79.1% (34)	79.1% (34)		1.00
Fissural cyst	97.7% (42)	97.7% (42)		1.00
Subpleural cyst	100.0% (43)	100.0% (43)		1.00
Encircling bronchovascular bundle (air-cuff sign)	62.8% (27)	79.1% (34)		*0.02*

Numbers within parenthesis indicate the number of patients

Statistics on the change in cyst size were calculated using the paired t-test after converting into 1-year change rate due to the difference between intervals according to CT

A paired t-test was performed for continuous variables, and exact McNemar's test was performed for categorical variables

The side-by-side comparison of the initial CT and final CT showed size progression in 48.8%, number progression in 37.2%, shape progression in 44.2%, and interstitial abnormality in 2.3% of patients between the two CTs. For cyst size, the median FU interval was 10.0 years in the progression group and 2.2 years in the stable group between the initial and final CTs. For cyst numbers, the median follow-up (FU) interval was 10.4 years in the progression group and 2.5 years in the stable group. For cyst shape, the median FU interval was 10.0 years in the progression group and 2.2 years in the stable group.

### Quantitative assessment of pulmonary cysts on thoracic CT

The average follow-up interval for initial CT and last CT was 3.8 years, and the median follow-up interval was 3.9 years in 17 patients. The mean and median values of LAA (%) of pulmonary cysts from 17 patients measured on the initial CT were 1.38% and 0.74% for each (range 0.06–4.99). The mean and median values of LAA (%) of pulmonary cysts from 17 patients measured on the last CT were 1.46% and 0.73% for each (range 0.12–5.00). The difference in LAA (%) between the initial and last CT was not statistically significant (*p* = 0.67).

**Fig. 1:**
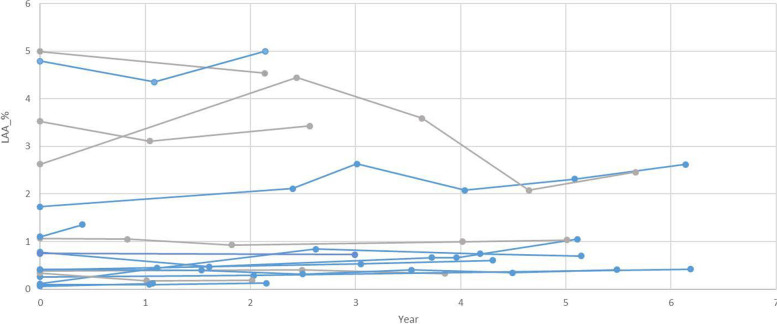
Result of quantitative assessment. Dot plots for the evolution of the volume of cysts in serial thoracic CT for 17 patients who had available 1-mm thin section CT data with serial follow up for at least 6 months. Eleven patients showed an interval increase in the LAA % between the initial and last CT (shown as blue lines). Six patients showed no significant interval change in the LAA % (shown as gray lines)

The quantification result of thoracic CT was calculated as the ratio of the cyst volume (LAA) to the total lung volume, and the raw data are plotted in Fig. 1. Eleven patients among a total of 17 patients showed interval increases in LAA % between the initial and last CT. Six patients among a total of 17 patients showed no definite increase or minimal decrease in LAA % on the last CT.

**Fig. 2:**
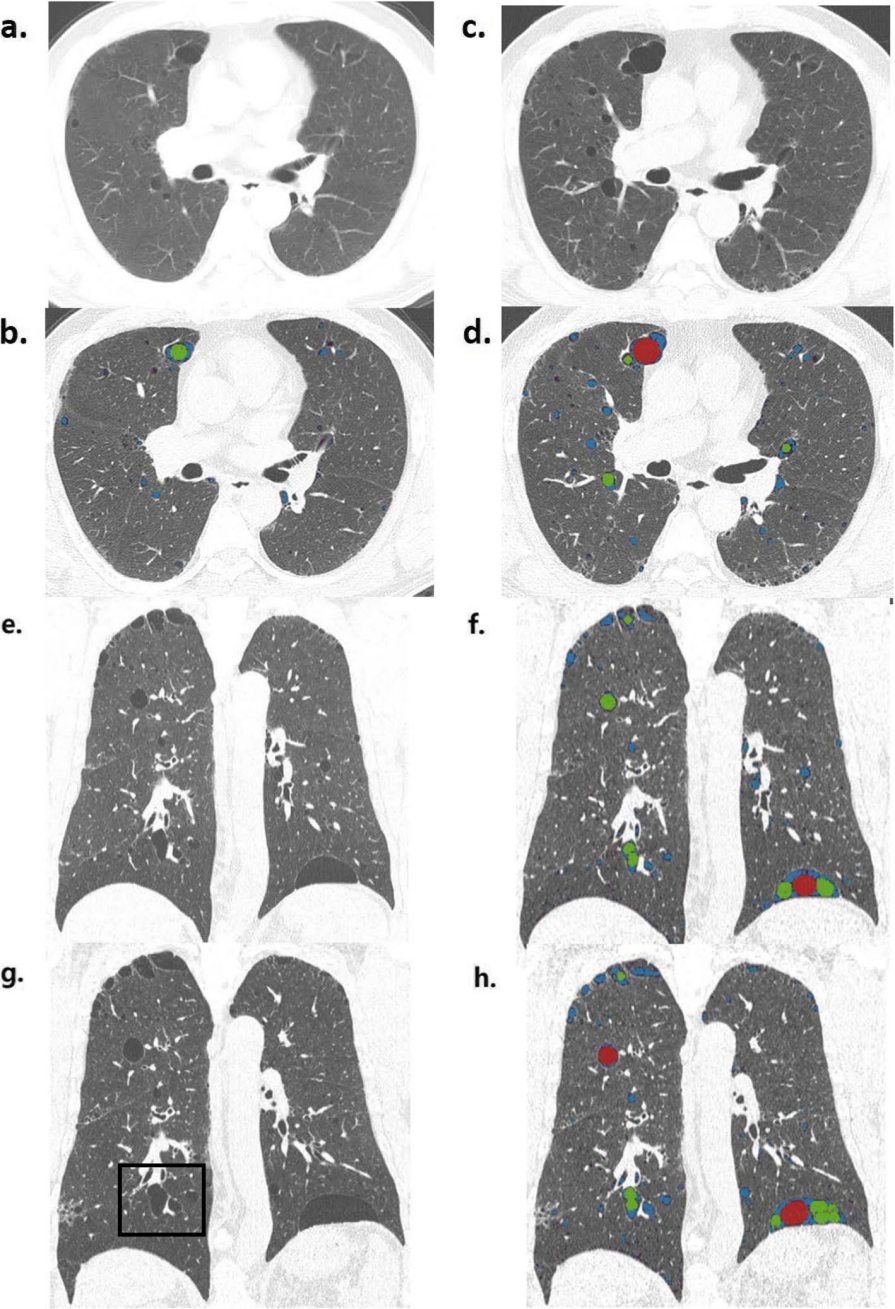
A representative case showing cyst progression in a 54-year-old male with BHD disease. **a**, **b**, **e**, **f** Baseline axial and coronal CT images and figures. **c**, **d**, **g**, **h** CT images obtained 6 years later. **b**, **d**, **f**, **h** Low attenuation area as a color map according to cyst size analyzed by in-house software. Black box in **g** indicates a cyst with “air-cuff signs”

As a representative case, Fig. 2 is a follow-up CT image at 6-year intervals of a 54-year-old male patient diagnosed with BHD. It can be seen in the image that the size and number of cysts at the same site increased at the same level on CT obtained 6 years later, and the results of displaying LAA using in-house software are also shown.

### Assessment of lung function by PFT

**Fig. 3:**
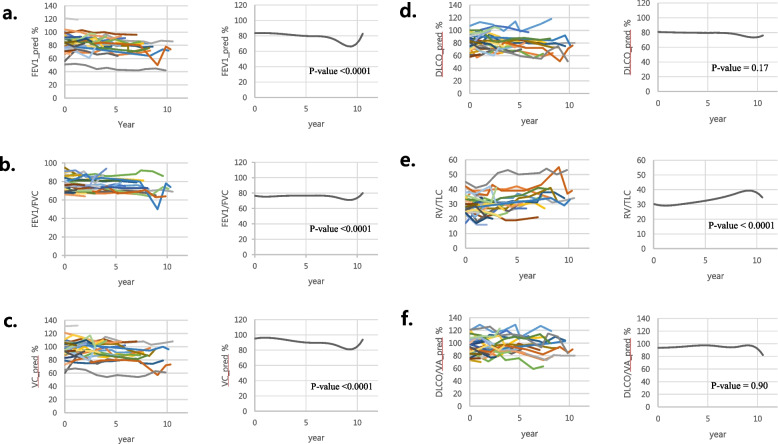
Results of the evolution of the PFT. Evolution of the parameters of the PFT during serial follow up in patients with BHD syndrome, left: raw data, right: linear mixed model, **a**) FEV1, **b**) FEV1/FVC, **c**) VC, **d**) DLCO, **e**) RV/TLC, and **f**) DLCO/VA. FEV1_pred %, FEV1/FVC, and VC pred_% among the PFT parameters showed a statistically significant decreasing trend with time (*p* < 0.0001 for each) and RV/TLC values showed a statistically significant increasing trend over time (*p* < 0.0001). DLCO and DLCO/VA values showed no significant change over time. FEV1 forced expiratory volume in one second, FVC forced vital capacity, TLC total lung capacity, RV residual volume, DLco carbon monoxide transfer factor, DLco/VA carbon monoxide transfer coefficient, %pred percentage of predicted value capacity

Thirty-three patients had available PFT with follow-up PFT over 6 months. The PFT values according to the follow-up period were investigated for six variables: FEV1pred%, FEV1/FVC, VCpred%, DL_CO_pred%, RV/TLC, and DL_CO_/Vapred%. The changes between the initial PFT and the final PFT were compared for statistical significance by calculating the respective rate of change, and the results are shown in Table 3. Although predicted FEV1 and FEV1/FVC showed a decreasing trend, there was no statistically significant change. The raw data according to the follow-up period of each variable were plotted, and the average value was plotted through spline by applying this to a mixed linear model. The plotted results are shown in Fig. 3. When the linear mixed model was applied by plotting all PFT data, the FEV1pred%, FEV1/FVC, and Vcpred%, among the PFT parameters, showed a statistically significant decreasing trend with time (*p* < 0.0001 for each). RV/TLC values showed a statistically significant increasing trend over time (*p* < 0.0001).

**Table 3:** Statistics of the differences in pulmonary function parameters of initial PFT and last PFT (*n* = 33)

	**Initial PFT**	**Last PFT**	**Difference**	***p*-value**
FEV1 pred(%)	84.0 ± 13.3	83.2 ± 13.1	-0.32 (-0.96, 0.32)	0.31
FEV1/FVC	78.6 ± 7.5	75.9 ± 7.1	-0.67 (-1.35, 0.01)	0.05
VC pred(%)	95.2 ± 14.9	95.5 ± 14.3	0.26 (-0.57, 1.08)	0.53
RV/TLC	30.1 ± 6.4	29.7 ± 6.2	-0.44 (-1.14, 0.25)	0.20
Dlco pred(%)	80.7 ± 11.6	81.2 ± 11.5	0.50 (-1.40, 2.41)	0.60
Dlco/VA	93.2 ± 14.2	93.3 ± 13.6	0.14 (-1.24, 1.52)	0.83

Statistics of the change in PFT were calculated using the paired t-test after converting into the 1-year change rate

### Risk factor analysis of pneumothorax

The results of binary logistic regression performed to investigate the risk factor for pneumothorax are presented in Table 4. Family history of pneumothorax was a factor related to the risk of pneumothorax (*p *= 0.002), whereas other factors including a history of pneumothorax did not show a clear association.

**Table 4:** Results of risk factor analysis of pneumothorax

	**OR**	**95% CI**		***p*-value**
		**lower**	**Upper**	
Family history of pneumothorax	19.84	2.98	132.12	*0.002*
History of pneumothorax	2.44	0.62	9.64	0.20
Maximal cyst size	0.98	0.94	1.03	0.49
Cyst number				
≤ 50	1.00			
50–100	0.66	0.15	2.97	0.67
≥ 100	0.97	0.08	11.24	0.89
Cyst shape				
Round/ovoid	1.00			
Lentiform	0.79	0.14	4.50	0.79
Irregular	0.11	0.01	2.67	0.18
Septated	1.90	0.11	32.66	0.66
Fissural cyst	2.92	0.07	127.04	0.58
Encircling bronchovascular bundle (air-cuff sign)	1.26	0.34	4.63	0.73
Location of largest cyst (subpleural)	1.20	0.25	5.83	0.82

## Discussion

It is not known whether pulmonary cysts in patients with BHD syndrome progress over time and whether pulmonary function declines during follow-up. Our study showed that the size of pulmonary cysts slightly progressed over time in longitudinal follow-up thoracic CT. The PFT also showed a slight deterioration in longitudinal follow-up PFT.

We performed semi-quantitative visual assessment in terms of size, number, shape, and other radiologic findings on the initial and last CT. We also performed a side-by-side comparison of the initial and last CT to determine the progression in terms of size, number, and shape. For the quantitative assessment of CT, the LAA % change was plotted along the longitudinal FU. PFT was also plotted along the longitudinal FU. Due to the inherent limitation of a rare disease, we did not obtain consistent results using several methods of assessment. However, the size of the largest cyst obtained by semi-quantitative assessment showed a significant interval increase, and the change in shape via the increasing incidence of cysts showing “air-cuff signs” was revealed as positive. For the side-by-side comparison, we identified many cases as progression in terms of size, number, and change in shape. For pulmonary function, the FEV1pred %, FEV1/FVC, and VC pred% showed a significant decrease at FU. These findings were not reported by previous studies examining the clinical course of BHD syndrome.

Pulmonary cysts of BHD disease have an irregular shape, large size, subpleural location, and air-cuff signs when compared with other cystic lung diseases such as lymphangioleiomyomatosis (LAM) [10]. In this study, as a result of analyzing the morphological features of initial thoracic CT, 86.0% (*n* = 37) of patients had irregularly shaped cysts, 95.3% (*n *= 41) of patients had fissural cysts, 100.0% (*n* = 43) of patients had subpleural cysts, and cysts with an air-cuff sign were seen in 62.8% (*n* = 27) of patients. These results correlated with the morphological features of the pulmonary cysts of BHD shown in previous studies.

However, few studies have investigated whether pulmonary cysts in patients with BHD syndrome progress over time after long-term follow-up. In one study, BHD syndrome, unlike LAM, did not show sufficient progression to cause respiratory failure but may have caused spontaneous pneumothorax, so conservative management may be necessary [12].

In the quantitative analysis, the average LAA % of the initial and last CT in 17 patients were 1.38% and 1.46%, respectively, and these values are in good agreement with the values ​​of 1.2–4.2% reported in the literature [22–24]. Also, although the difference was insignificant, the mean value was slightly increased in the initial and last CT. The LAA % of the serial follow up thoracic CT showed a trend to be mildly increased.

According to other studies, the pulmonary function in patients with BHD syndrome does not change significantly during the follow-up period [11, 19]. In the present study, the six parameters we measured did not show a statistically significant difference when simply comparing the initial and final PFTs. However, when the linear mixed model was applied by plotting all PFT data, the FEV1pred %, FEV1/FVC, and VC pred% among the PFT parameters showed a statistically significant decreasing trend with time (*p* < 0.0001 for each) and RV/TLC values showed a statistically significant increasing trend over time (*p* < 0.0001). Results revealed by a linear mixed model might reflect the interval change more precisely because the analysis included all the data of FU PFT. However, we sought to investigate whether PFT progresses via regular and long-term FU PFT in a prospective study. Although this was mild, the long-term continuous follow-up of patients with BHD syndrome may lead to a deterioration in pulmonary function, which may have an important effect on the prognosis and quality of life in patients with BHD syndrome.

Pneumothorax can be life-threatening and may affect the quality of life of patients with BHD syndrome. According to a study by Toro et al., the cumulative risk of having the first pneumothorax is 75% by age 50 years [17]. Performing pleurodesis as soon as the first spontaneous pneumothorax is detected prevents recurrent episodes [6]. In our study, 51% of the patients developed pneumothorax, of whom 28% showed recurrent pneumothorax. Previous studies showed the number of cysts, size of cysts, maximum size of cysts, and volume of cysts were risk factors for pneumothorax [4, 10, 17, 18]. In our study, the size of cysts or number of cysts were not revealed as risk factors. The number of cysts was analyzed and categorized into groups (≤ 50, 50 ~ 100, and ≥ 100), which might have been a limitation of analysis because the number of enrolled subjects was small and consequently the statistical power was low. Interestingly, only a family history of pneumothorax was identified as a risk factor in our study. In the study by Sattler et al., several specific mutations among FLCN mutations (c.1300G>C and c.250-2A>G) showed significantly increased risks for pneumothorax compared with patients with other types of FLCN mutations [25]. This could explain the familial character of pneumothorax that appeared in our study.

Many cysts located along the pleura in patients with BHD syndrome might be vulnerable to rupture and consequent pneumothorax. Several pathology studies reported the histopathologic features of pulmonary cysts, which expanded toward the visceral pleura and were partially embedded in an interlobular septum and bronchovascular bundles [26–28]. This location is well correlated with the cyst location under the pleura and around the bronchovascular bundle seen on thoracic CT. In addition to location, pathologically abnormal epithelial/mesenchymal interactions induced by FLCN mutation may weaken the extracellular matrix of the visceral pleura, leading to pneumothorax [5]. In a study by Toro et al., the number of cysts located on the pleural surface was a risk factor for pneumothorax [17]. However, the subpleural location of cysts was not a risk factor in our study.

Although the mechanism of pulmonary cyst formation is not well understood, it is known to be associated with the folliculin gene. According to a study of the pathology of pulmonary cysts in Japanese patients with BHD syndrome [9], a possible mechanism might be *FLCN* gene loss in the lung epithelium, which could cause changes in epithelial adhesion and survival and induce alveolar enlargement. This theory is similar to the previously known “stretch mechanism”, which reasons that pulmonary cysts occur due to stretch-induced stress [29]. If pulmonary cysts occur in patients with BHD syndrome by this mechanism, pulmonary cysts should increase slowly over time.

There were several limitations in this study. It was a single-center retrospective study, so it has an inherent limitation wherein the results may not reflect the entire BHD patient group. In addition, this study was conducted with 43 patients, and there was a difference in the follow-up period for each patient. Using 6-month FU CT as an inclusion criterion was too short to evaluate changes in cysts. However, our study included more patients compared with previous studies on BHD—which is a very rare disease—and includes long-term follow-up data.

In conclusion, pulmonary cysts mildly progressed in size over time in patients with BHD syndrome. Pulmonary function showed a mild deterioration in the longitudinal follow-up. In addition, a family history of pneumothorax was a risk factor for pneumothorax. Therefore, the long-term follow-up of pulmonary cysts in patients with BHD could improve the management of patients and their quality of life.

## Data Availability

The data that support the findings of this study are available from the Asan Medical Center, but restrictions apply to the availability of these data, which were used for the current study, and so are not publicly available. Data are however available from the authors upon reasonable request and with permission of the Asan Medical Center.

## Abbreviations

BHD: Birt-Hogg-Dubé syndrome
CT: Computed tomography
PFT: Pulmonary function test
EMR: Electronic medical record
TLC: Total lung capacity
FEV1: Forced expiratory volume in 1 second
FEV1/FVC: Ratio of FEV1 to forced vital capacity
VC: Vital capacity
RV/TLC: Ratio of residual volume to total lung capacity
DLco: Diffusing capacity of the lung for carbon monoxide
DLco/VA: Ratio of DLco to alveolar volume
LAA: Low attenuation area
